# Early mortality from external causes in Aboriginal mothers: a retrospective cohort study

**DOI:** 10.1186/s12889-016-3101-2

**Published:** 2016-06-01

**Authors:** Jenny Fairthorne, Roz Walker, Nick de Klerk, Carrington Shepherd

**Affiliations:** Telethon Kids Institute, University of Western Australia, Perth, Australia; Department of Paediatrics, Child and Family Research Institute, University of British Columbia, Vancouver, Canada

**Keywords:** Aboriginal, Mother, Death, Linked data, Homicide, Suicide, Accident

## Abstract

**Background:**

Maternal loss can have a deep-rooted impact on families. Whilst a disproportionate number of Aboriginal women die from potentially preventable causes, no research has investigated mortality in Aboriginal mothers. We aimed to examine the elevated mortality risk in Aboriginal mothers with a focus on external causes.

**Methods:**

We linked data from four state administrative datasets to identify all women who had a child from 1983 to 2010 in Western Australia and ascertained their Aboriginality, socio-demographic details, and their dates and causes of death prior to 2011. Comparing Aboriginal mothers with other mothers, we estimated the hazard ratios (HRs) for death by any external cause and each of the sub-categories of accident, suicide, and homicide, and the corresponding age of their youngest child.

**Results:**

Compared to non-Aboriginal mothers and after adjustment for parity, socio-economic status and remoteness, Aboriginal mothers were more likely to die from accidents [HR = 6.43 (95 % CI: 4.9, 8.4)], suicide [HR = 3.46 (95 % CI: 2.2, 5.4)], homicide [HR = 17.46 (95 % CI: 10.4, 29.2)] or any external cause [HR = 6.61 (95 % CI: 5.4, 8.1)]. For mothers experiencing death, the median age of their youngest child was 4.8 years.

**Conclusion:**

During the study period, Aboriginal mothers were much more likely to die than other mothers and they usually left more and younger children. These increased rates were only partly explained by socio-demographic circumstances. Further research is required to examine the risk factors associated with these potentially preventable deaths and to enable the development of informed health promotion to increase the life chances of Aboriginal mothers and their children.

**Electronic supplementary material:**

The online version of this article (doi:10.1186/s12889-016-3101-2) contains supplementary material, which is available to authorized users.

## Background

Australia’s Aboriginal and Torres Strait Islander peoples (henceforth referred to as *Aboriginal*) have poorer health and lower life expectancy than other Australians [1]. The disparity in life expectancy estimates are in the order of nine to eleven years [1], reflecting higher mortality rates at every stage of the life course [2, 3]. The reasons are not fully understood, although they are likely to include a complex mix of social determinants, behavioural and other risk factors [1].

Lower life expectancy, coupled with higher fertility rates, gives rise to a younger age profile in Aboriginal Australia [4]. Consequently, Aboriginal children have fewer older and experienced people available for care, protection, cultural guidance and as teachers of general life skills. Aboriginal mothers (and grandmothers) are especially important in this regard, as they are typically the primary caregivers and have the most influence on the cultural, social and spiritual health of children [5] and thus the health of subsequent generations. We know that the mortality rates due to external causes are higher in Aboriginal than non-Aboriginal people. For example, land transport accidents, along with suicide were leading causes of death for Aboriginal people in 2013 with the age-standardised rate being more than twice that of non-Aboriginal Australians [6]. Aboriginal women also have higher rates of death due to external causes and higher rates in the sub-categories of accident, suicide and homicide than other women [6]. In particular, transport accidents and suicide are among the most frequent causes of deaths and are from two to three times the rate for non-Aboriginal women [1, 7]. Furthermore, the majority of suicides in Australian Aboriginal people are due to hanging which has implications for prevention strategies [8–10]. Such events are particularly traumatic for children and negatively impact their later development. For example, higher rates of substance abuse were identified in children who had experienced adverse child experiences [11] and more specifically, Aboriginal children who had experienced the death of their mother suffered higher rates of both substance abuse and suicide [12].

A mother’s health and presence in the family are important predictors of her children’s health [12, 13] and deaths can have a deep-rooted impact on families and communities. However, no study has quantified the extent of these deaths amongst Aboriginal mothers and we found no study which produced rates adjusted for likely confounders and compared to non-Aboriginal mothers. Deaths due to external causes are largely preventable. Thus, an understanding of these events and the development of informed initiatives aimed at prevention would likely result in a timely reduction in their occurrence. In turn, this would benefit the health of Aboriginal children in the current and subsequent generations. Accordingly, the purpose of this study was to examine differences in the mortality risks between Aboriginal and non-Aboriginal mothers in Western Australia (WA) and the number and ages of affected children. Specifically, we aimed to:

1. Estimate survival rates in Aboriginal mothers compared with non-Aboriginal mothers;
2. Describe the ages of maternal deaths and the ages of the youngest child by cause of death;
3. Estimate the number of children who experienced the loss of their mother and the corresponding size of affected families;
4. Estimate hazard ratios (HRs) for death by external cause, adjusting for maternal age, socioeconomic status (SES), parity, geographic remoteness, and year of birth of the youngest child; and
5. Compare the HRs over the three decades of the study period.

## Methods

Our data were obtained from four WA state-wide sources. From the *Midwives Notification System* (MNS), we identified our study population which was all women who had a live-born child between 1983 and 2010, and extracted various socio-demographic traits, a binary measure of Aboriginality, maternal birth date and the birth date of the youngest live-born child for each mother. From the *Death Registry*, we ascertained the dates and causes of death of women in this group who had died before 2011 and two other variables measuring Aboriginality. Lastly, we accessed data-sets from the *Hospital Morbidity Data System* (HMDS) and the *Mental Health Information System* where we obtained a variable from each which also measured maternal Aboriginality. These data sources have been shown to be extremely reliable and feature high levels of completeness among core data items [14]. We linked all data-sets using a unique alpha-numeric identifier created by the *Data Linkage Branch* (DLB) [15] after probabilistic record linkage of the four datasets. The methods used by the DLB are internationally accepted as best practice and result in very few incorrect links [14, 16].

### Aboriginal status

Five variables (from four datasets) were used to identify the Aboriginal status of mothers in the study sample. We used a two-step process, following the guidelines of the *Getting Our Story Right* project [17, 18], to ascertain Aboriginal mothers. First, Aboriginal status is determined within each dataset, and in the WA Death Registry and HMDS, this status is based on multiple indicators. Second, an overall derived Aboriginal status was calculated for each study mother, by summing the derived information on Aboriginal status across all datasets. The same decision rule was applied to both steps: if there were two or more Aboriginal identifiers then the person was considered Aboriginal for the purposes of this study; if only one or two identifiers were available then Aboriginal status was derived if at least one identified them as Aboriginal [17, 18].

### Index child and maternal groups

The index child of a woman was her youngest live-born child born from 1st January, 1983 to 31st December, 2010. The case group consisted of all *Aboriginal* women with a child born during the study period. The comparator group consisted of all women who were assessed as *non-Aboriginal* and with a child born during the same period.

### Explanatory variables

Parity [19], socio-economic status [20, 21] and remoteness [20, 21] are recognised confounders of the association between Aboriginality and health outcomes. Hence, when calculating the HRs, we included these traits in a multivariate model. In order to account for possible changes in mortality rates, we also included the year group of the index birth in this model. The variable for maternal age was the age of the mother at the time of the index birth and her parity was the number of previous live-births at this time. Socio-economic status was determined from quintiles of the *Indices of Relative Socioeconomic Disadvantage* [22] for 2001. This uses residence grouped by the unit termed ‘collection district’. For mothers where this was not available, we used the same index but with measures from 1996 or 2006 or a similar index for 2001 which used ‘Statistical local areas’ which was larger than collection districts. If values were missing at the time of the index birth, we used the measures which were available at the closest previous birth of the mother. The quintiles were labelled ‘very high’ ‘high’ ‘medium’ ‘low’ and ‘disadvantaged’ in descending order. Initially, our covariate for remoteness used the *Accessibility/Remoteness Index of Australia* (ARIA) data based on collection districts for 2006 [22]. At an individual level, if this was unavailable, we used the corresponding values for 2001 and then 1996. Where, no collection district data was available, we used ARIA values for Statistical Local Areas in the same way. In the event that ARIA values were not available at either of these levels, we used the ARIA values for Local Government Areas.

### ICD cause of death codes

We used ICD-9 and ICD-10 cause of death codes to group the external causes of death into the five categories of: *Accidents* and the sub-category of *Transport accidents*; *Suicide* and the sub-category of *Hanging*; *Homicide*; *Other external causes* and *Any external cause*. ICD-9 codes were used in WA prior to 1999 and ICD-10 codes thereafter [23]. The codes relating to these previous groupings are provided in Additional file 1: Table S1. These groups were modified from the cause of death codes described by the World Health Organisation [24] and the Centers for Disease Control and Prevention [25]. Due to the unreliability of analyses in groups where the sample size is less than seven [26], analyses were only performed on categories containing seven or more mothers.

### Analyses

To plot the survival curves of Aboriginal and non-Aboriginal mothers, we used the Kaplan-Meier estimator. Numbers permitting, we used Cox Proportional Hazards Regression with death by *any external cause, accident*, *transport accident*, *suicide*, *hanging*, *homicide* and *other external causes* as the events of interest. The underlying time variable was the number of years from the date of birth of her last child born between January 1, 1983 and 31st December, 2010 to the date of her death or 31st December, 2010, whichever was earlier. We compared the Aboriginal mothers to the non-Aboriginal mothers and calculated the univariate and multivariate HRs (with the explanatory variables described above) for each of the seven categories of death. To assess whether the HRs had changed over time, we divided the study period into shorter periods of; *1983*–*1990*, *1991*–*2000* and *2001*–*2010*. For each period, mothers entered the study only if they had not died in an earlier period and their youngest child was born in or before that period. The entry point was their youngest child’s date of birth or the start of the period, whichever was later, and exited at the end of that period or their date of death, whichever was earlier. Because of smaller numbers, we then performed two separate multivariate analyses for each of all external causes and all accidents within each calendar period. To compare the age of non-Aboriginal and Aboriginal mothers at the time of death, we calculated the median age, upper and lower quartiles of age at death by our cause of death categories. We also calculated the median age of the youngest children at the time of maternal death by these cause of death categories.

We estimated the number of surviving children of the deceased mothers according to their Aboriginal status. We used the proxy of *index parity plus one* as an estimate of the number of surviving children for each deceased Aboriginal (Est_AD_) and non-Aboriginal mother (Est_ND_) and then summed the estimates for Aboriginal mothers (∑Est_AD_) and non-Aboriginal mothers (∑Est_1ND_). In a similar way, we estimated the total number of surviving children of surviving mothers according to their Aboriginal status (∑Est_1AS_ and ∑Est_1NS_). We then estimated the proportion of children who experienced maternal death according to maternal Aboriginal status by dividing the estimates for the Aboriginal group (∑Est_1AD_) by the estimated total number of surviving children of all Aboriginal mothers (∑Est_1AD_ + ∑ Est_1AS_) and in the same way for the non-Aboriginal group (∑Est_1ND_/[∑Est_1ND +_ ∑Est_1NS_]). Finally, we compared the mean parity of Aboriginal women who had experienced death to the parity of Aboriginal women who had not. All proportions were compared using a chi-square for proportions and STATA 13 was used for all analyses.

## Results

### Socio-demographic factors

About 4 % of mothers were Aboriginal and these mothers were much more likely to die from an external cause or from any cause than non-Aboriginal mothers. All the demographic covariates had strikingly different distributions in the Aboriginal mothers from those in the non-Aboriginal mothers (Table 1).

**Table 1:** Numbers (%) of mothers by cause of death, demographics and Aboriginality

Variable	Aboriginal	Non-Aboriginal	Total
Cause of death			
External cause^a^	197 (1.3 %)	668 (0.2 %)	865 (0.2 %)
Other causes^b^	517 (3.3 %)	2,396 (0.7 %)	2,913 (0.8 %)
All cause death	714 (4.6 %)	3,064 (0.9 %)	3,778 (1.0 %)
Aboriginal status			
	15, 606 (4.2 %)	358,239 (95.8 %)	373,845 (100 %)
Socio-economic status by quintile			
Missing	964 (6.2 %)	14,344 (4.0 %)	15,308 (4.1 %)
Very high	384 (2.5 %)	76,588 (21.4 %)	76,972 (20.6 %)
High	934 (6.0 %)	72,629 (20.3 %)	73,565 (19.7 %)
Medium	1,568 (10.1 %)	72,512 (20.2 %)	74,080(19.8 %)
Low	2,698 (17.3 %)	68,385 (19.2 %)	71,533 (19.1 %)
Disadvantaged	9,058 (58.0 %)	53,331 (14.9 %)	62, 389 (16.7 %)
Maternal age (in years) at the index birth			
Less than 15	96 (0.6 %)	47 (0.01 %)	143 (0.04 %)
15 & < 20	2,314 (14.8 %)	8,643 (2.4 %)	10,957 (2.9 %)
20 & < 25	4,317 (27.7 %)	42,614 (11.9 %)	46,931 (12.6 %)
26 & < 30	4,225(27.1 %)	103,098 (28.8 %)	107,323 (28.7 %)
31 & < 35	2,914 (18.7 %)	124,438 (34.7 %)	127,352 (34.1 %)
36 & < 40	1,444 (9.3 %)	66,072 (18.4 %)	67,516 (18.1 %)
40 & over	296 (1.9 %)	13,327 (3.7 %)	13,623 (3.6 %)
Parity at the index birth			
No previous child	3,182 (20.4 %)	88,766 (24.8 %)	91,948 (24.6 %)
One previous child	3,108 (19.9 %)	146,281 (40.8 %)	149,389 (40.0 %)
2-3 previous children	5,451 (34.9 %)	109,167 (30.5 %)	114,618 (30.7 %)
4-6 previous children	3,284 (21.0 %)	13,210 (3.7 %)	16,494 (4.4 %)
>7 previous children	581 (3.7 %)	815 (0.2 %)	1,396 (0.4 %)
Remoteness			
Missing	689 (4.4 %)	9,242 (2.6 %)	9,931 (2.7 %)
Urban	3,642 (23.3 %)	227,002 (63.4 %)	230,644 (61.7 %)
Rural/outer urban	1,614 (10.3 %)	62,440 (17.4 %)	64,054 (17.1)
Distant	1,886 (12.1 %)	27,970 (7.8 %)	29,856 (8.0 %)
Remote	2,079 (13.3 %)	16,041 (4.5 %)	18,120 (4.9 %)
Very remote	5,696 (36.5 %)	15,544 (4.3 %)	21,240 (5.7 %)
Year of birth of index child			
1983–1986	1,231 (7.9 %)	37,641 (10.5 %)	38,872 (10.4 %)
1987–1990	1,544 (9.9 %)	41,939 (11.7 %)	43,483 (11.6 %)
1991–1994	1,463 (9.4 %)	43,035 (12.0 %)	44,498 (11.9 %)
1995–1998	1,580 (10.1 %)	44,928 (12.5 %)	46,508 (12.4 %)
1999–2002	1,915 (12.3 %)	45,010 (12.6 %)	46,925 (12.6 %)
2003–2006	2,440 (15.6 %)	50,418 (14.1 %)	52,858 (14.1 %)
2007–2010	5,433 (34.8 %)	95,268 (26.6 %)	100,701 (26.9 %)
Total	15,606 (100 %)	358,239 (100 %)	373,845 (100 %)

^a^External cause: Associated ICD-9 and 10 codes are listed in Additional file 1: Table S1

^b^ *Other causes* refers to death with an associated code from ICD-9 codes: E870-E879, E930-E949, E980-E984, E985.5-E998 or ICD-10 codes: Y10-Y84, Y87.2-Y98 [20]

### Survival rates

**Fig. 1:**
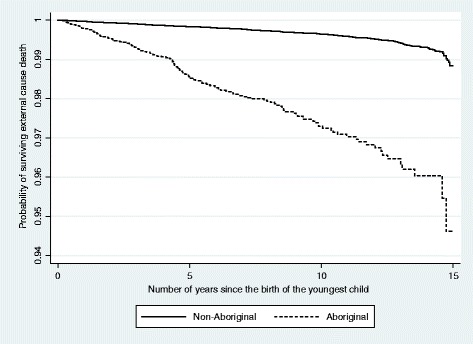
Maternal survival of external cause death by Aboriginality. Note: The y-axis is truncated at 0.94

The survival of Aboriginal mothers was significantly lower (log-rank p-value  < 0.00005) than non-Aboriginal mothers and at fifteen years after the index birth, the survival rate of Aboriginal mothers was about 95 % compared with about 99 % for non-Aboriginal mothers (Fig. 1).

### Hazard ratios by cause of death category

The category, *other external causes* was excluded from further analysis as there were only six Aboriginal mothers who died from a cause within this category (Table 2). Accidents were the most common external cause of death in Aboriginal mothers (*n* = 116) and the most common type was transport accidents (*n* = 78). Homicide was the next most common external cause (*n* = 50) with more Aboriginal mothers experiencing death by homicide than non-Aboriginal mothers (Table 2).

**Table 2:** Number (%) of mothers by Aboriginal status and cause of death by category and major sub-categories

Cause of death	Aboriginal	non-Aboriginal	Total
Any external cause	197 (1.3)	668 (0.19)	865 (0.23)
1. Accident	116 (0.7)	368 (0.10)	484 (0.1)
Transport accidents	78 (0.49)	177 (0.05)	255 (0.07)
2. Suicide	27 (0.2)	244 (0.07)	271 (0.07)
Hanging	18 (0.12)	88 (0.02)	106 (0.03)
3. Homicide	50 (0.32)	44 (0.01)	94 (0.03)
4. Other external causes	6 (0.04)	16 (0.00)	22 (0.01)

Note: Percentages are of total numbers of Aboriginal (*N* = 15,606) and non-Aboriginal (*N* = 358,239) mothers

The adjusted relative rates or HRs comparing Aboriginal with non-Aboriginal mothers were between 3.5 and 17.5 for each cause of death group, and all p-values were less than 0.0005 (Table 3). Compared to non-Aboriginal mothers, Aboriginal mothers were about 6.5 times more likely to die as a result of an accident [HR = 6.43 (95 % CI: 4.9, 8.4)], particularly transport accidents [HR = 7.01 ((95 % CI: 5.0, 9.9)], about 3.5 times more likely to die from suicide [HR = 3.46 (95 % CI: 2.2, 5.4)] and more than five times as likely to complete suicide by hanging [HR = 5.28 (95 % CI: 2.9, 9.8)]. Aboriginal mothers were about 17.5 times more likely to die from homicide [HR = 17.46 (95 % CI: 10.4, 29.2)] and overall, more than 6.5 times as likely to die from any external cause than non-Aboriginal mothers [HR = 6.61 (95 % CI: 5.4, 8.1)] (Table 3).

**Table 3:** Univariate and multivariate HRs for death in mothers by cause and Aboriginal status

Cause of death	Non-Aboriginal (comparator)	Aboriginal	
		Univariate	Multivariate
Accidents	1	8.79 (7.1, 10.8)	6.43 (4.9, 8.4)
Transport	1	12.4 (9.5, 16.2)	7.01 (5.0, 9.9)
Suicide	1	3.06 (2.1, 4.6)	3.46 (2.2, 5.4)
Hanging	1	5.62 (3.4, 9.3)	5.28 (2.9, 9.8)
Homicide	1	32.11 (21.4, 48.2)	17.46 (10.4, 29.2)
Any external cause	1	8.20 (7.0, 9.6)	6.61 (5.4, 8.1)

All *p*-values for difference in hazard rates <0.0005

The multivariate model includes age of mother, SES, parity, remoteness, and calendar year measured at the time of the index birth

### Changes over time

For Period 1 and Period 2, the multivariate HRs for accidental death for Aboriginal compared to non-Aboriginal mothers changed only slightly [HR = 4.35 (95 % CI: 3.3, 5.7), HR = 4.26 (95 % CI: 3.2, 5.6)] but in Period 3, the adjusted HRs rose considerably [HR = 7.13 (5.1, 10.0)]. For death by any external cause, the multivariate HR remained almost constant [HR = 4.48 (95 % CI: 3.7, 5.5), HR = 4.48 (95 % CI: 3.6, 5.5)]. In Period 3, the HR again increased [HR = 6.90(5.3, 8.9)] (Table 4).

**Table 4:** Univariate and multivariate HRs for death by accident and death by any external cause in mothers by Aboriginal status and time period of death

Cause of death	Time period		Non-Aboriginal (comparator)	Aboriginal	
				Univariate	Multivariate
Accident	Period 1	1983–1990	1	7.24(5.8, 8.9)	4.35(3.3, 5.7)
	Period 2	1991–2000	1	7.05(5.7, 8.8)	4.26(3.2, 5.6)
	Period 3	2001–2010	1	9.64(7.4, 12.6)	7.13(5.1, 10.0)
Any external cause	Period 1	1983–1990	1	6.78 (5.8,7.9)	4.48(3.7, 5.5)
	Period 2	1991–2000	1	6.67(5.6, 7.9)	4.48(3.6, 5.5)
	Period 3	2001–2010	1	8.29(6.8, 10.2)	6.90(5.3, 8.9)

All *p*-values <0.0005

### Maternal age at death

The median age of death by any external cause for deceased Aboriginal mothers was 33 years compared to 36.5 years in deceased non-Aboriginal mothers and this younger age for deceased Aboriginal mothers was found in all of the sub-categories (Table 5).

**Table 5:** Median age (upper and lower quartiles) of mothers at death by cause and Aboriginal status

Cause of death	Aboriginal	Non-Aboriginal	Overall
Accident			
Median age (25^th^, 75^th^) percentiles	33 (25, 45) years	36 (29.5, 43) years	36(29, 42) years
Transport accidents			
Median age (25^th^, 75^th^) percentiles	32.5 (23, 40) years	33 (29, 40) years	33 (27, 40) years
Suicide			
Median age (25^th^, 75^th^) percentiles	32 (28, 40) years	37 (31, 43) years	37 (26, 48) years
Hanging			
Median age (25^th^, 75^th^) percentiles	30.5 (27, 37) years	35.5 (30, 42.5) years	34 (29, 41) years
Homicide			
Median age (25^th^, 75^th^) percentiles	33 (21, 39) years	35.5 (22, 40) years	34 (25, 39) years
Any external cause			
Median age (25^th^, 75^th^) percentiles	33 (25, 40) years	36.5 (30, 43) years	36 (29, 42) years

### Age and number of children at mother’s death

For Aboriginal mothers, the median age of the youngest child at the time of the mother’s fatal accident was 5.3 years and 5.0 years for *transport accidents*. Where suicide was the cause of death, the median age of the youngest child was 4.4 years and for the sub-category of *hanging,* also 4.4 years. At the time of the maternal homicide, the median age of the child was 4.5 years and for the combined category of *any external cause*, the median age was 4.8 years.

For non-Aboriginal mothers who died of an accident, the median age of the youngest child was 6.9 years and 5.8 for *transport accidents*. At the time of maternal death by suicide in non-Aboriginal mothers, the median age of the youngest child was 7.3 years and for *hanging,* 7.1 years. At the homicide of a non-Aboriginal mother, the median age of the youngest child was 5.2 years and for the combined category of *any external cause*, the median age was 7.0 years (Table 6).

**Table 6:** Median age of youngest child (in years) by cause of maternal death and Aboriginal status

Cause of death	Aboriginal	Non-Aboriginal	Overall
Any external cause	4.8	7.0	6.4
Accident	5.3	6.9	6.6
Transport	5.0	5.8	5.5
Suicide	4.4	7.3	7.0
Hanging	4.4	7.1	6.1
Homicide	4.5	5.2	4.9

There were an estimated 17,561 surviving children of Aboriginal mothers and of these, 2,669 (15.2 %) experienced the death of their mother. Of the estimated 816,376 children of non-Aboriginal mothers, 7,735 (0.95 %) experienced the death of their mother) The Aboriginal children who experienced the loss of their mother came from an average family size of 3.74 children compared to 3.31 for Aboriginal children who had not experienced this loss (*p*-value <0.005). The non-Aboriginal children who experienced the loss of their mother came from an average family size of 2.52 children compared to 2.28 for those who had not experienced this loss (*p*-value <0.005).

At the time of the index birth, the mean parity of Aboriginal women who experienced death during the study period was 2.75 which was significantly more than the mean parity of 2.34 for Aboriginal women who did not experience death (p-value < 0.00005).

## Discussion

The study confirms that Aboriginal mothers are much more likely to die from external causes than non-Aboriginal mothers. This disparity exists for all external cause categories, with the scale of difference (as measured by the multivariate hazard ratios) between Aboriginal and non-Aboriginal mothers ranging from 3.5 times for suicide to nearly 17.5 times for homicide. Comparisons with the extant literature suggest that the magnitude of ethnic disparities in mortality from external causes is greater among mothers than the female population overall, identifying Aboriginal mothers as a particularly at-risk group. Moreover, the data indicate that the relative risks have increased over the 30-year period to 2010. Recent government reporting featured a decline in mortality among Aboriginal people due to disease, but no change in the rate of death from external causes [27]. Clearly, this report camouflages the increased maternal relative risk found in our data.

A substantially larger proportion of children born to Aboriginal mothers had experienced a maternal loss (5.4 %) when compared with other children (1.0 %). The data also indicated that, on average, children born to Aboriginal mothers suffer this loss at an earlier age, and often in the formative years of development (first five years of life). This is a period when children are particularly vulnerable, and where the circumstances leading to and arising from a maternal loss can have profoundly negative consequences for social and emotional wellbeing—including prolonged periods of grief, depression, stress, anxiety, problems with identity development, and the difficulties associated with the transition to out-of-home care [13, 28]. Further, studies on the impact of life stress, including parental loss, highlight that trauma in early life can also lead to onward problems with substance abuse, self-harming, suicide, anti-social behaviour, and other adversities into adulthood [12, 29]. The findings of Zubrick et al. (2011) suggest that maternal loss may subject Aboriginal children to additional developmental risk, above and beyond those posed by previously experienced high levels of life adversity [12]. This affirms our understanding that Aboriginal mothers typically have a central role in the cultural and spiritual development of Aboriginal children [30] in addition to being the primary caregiver.

Accidents were the most common external cause of death in both Aboriginal and non-Aboriginal mothers with transport accidents being the most frequent single cause. Aboriginal mothers were at about a six and a half times greater risk of death by accidents. Evidence suggests that differences in accidents and injuries are influenced by unequal access to resources that make environments safer [31]. In Aboriginal communities this is likely to extend to the quality of the built environment (including housing infrastructure and facilities, size and suitability, and roads), which is often cited as being sub-standard—particularly in remote areas [32]. The increased risk of transport accident deaths among Aboriginal mothers is likely to reflect a complex range of environmental, human and cultural factors [33]. Aboriginal Australians live in remote regions that typically have poorer roads and higher speed limits, and less access to vehicles which are safe in the environment in which they are used. The literature also suggests that Aboriginal people have an increased likelihood of travel in an emergency situation, a reduced likelihood of driver training, and higher levels of non-compliance with road laws (such as alcohol use and seatbelt wearing) [34]. Reducing transport accidents and associated fatalities in Aboriginal populations is a particularly pressing issue given the scale of deaths from this cause. Prevention and management programs that address the root causes of these deaths will have an impact on the disparities in maternal loss in Aboriginal and non-Aboriginal populations.

Our findings are consistent with Australian data for females, and support a two to three-fold increased risk of suicide for Aboriginal mothers when compared with other mothers [35]. Despite measurement difficulties, there is evidence that suggests there was a disproportionate increase in Aboriginal suicide rates in Australia after the 1980s [36] ─ the rates (and disparities) appear to have stabilised at the national level but there is still considerable variation by region [37]. The risk factors for suicide in Aboriginal people include a lack of purpose and role models [38], and dislocation from community and family ties [39]. For Aboriginal women specifically, higher suicide rates are associated with sexual abuse [40, 41] and especially intimate partner abuse [41]. Other associations include ongoing grief [39] and substance misuse [39]. The finding in this study of elevated suicide risks in Aboriginal mothers confirms the need for increased services to protect these women from abuse and domestic violence. Suggested initiatives are at the individual, extended family and community level which aim to develop networks [42] and foster healing in relation to the cumulative, inter-generational and pervasive effects of colonisation [43].

Homicide had the highest relative risk for Aboriginal mothers (17.5 times higher than non-Aboriginal mothers) over the study period, even after adjustment for known confounders. Notwithstanding, homicide remains an uncommon cause of death among both populations. The elevated risks for Aboriginal women are higher than expected given the six-fold increased univariate rates reported for the overall Aboriginal population in 2014 [42]. This may, in part, reflect the added burden of domestic violence issues for Aboriginal women and particularly Aboriginal mothers. In 1993, Easteal [44] noted that almost a half of the Aboriginal homicide victims were perpetrated by their partners, and at double the rate in non-Aboriginal victims. Other reports indicated that a majority of homicides are likely to involve husband and wife. For example, a report from the Australian Institute of Criminology described that while Aboriginal people were only 2 % of the Australian population, 25 % of intimate partner homicides were between Aboriginal people [45]. Further, Aboriginal people experience higher rates of family violence; particularly in remote areas where the rate of violence is 36 times higher [42]. A second risk factor for the higher homicide rate in Aboriginal mothers may be the consumption of alcohol. Homicides involving Aboriginal people commonly involve a high level of alcohol [46] and the majority are associated with alcohol consumption of both the victim and perpetrator [42]. High homicide rates among Aboriginal women appear to be a long-standing phenomenon—in 1987, nearly 80 % of all “deaths involving chargeable offences” in the Northern Territory were of Aboriginal women [47].

Our results consistently indicate that adjusting for socio-demographic factors partially attenuates the elevated risk of external cause death among Aboriginal mothers. We note from our data that Aboriginal mothers who died tended to be younger and have more children. Young mothers often face more difficult social and economic circumstances and receive insufficient support [48]. These factors can be stressful, put pressures on family functioning and limit the wherewithal to raise children, particularly in families with multiple children. These issues may be compounded in Aboriginal families if there are additional pressures to care for children of extended family members, and plausibly place Aboriginal mothers at greater risk of a range of accidents and injuries. Strategies that support young mothers and enhance their resilience and parenting skills and/or delay the onset of first pregnancy may prompt a reduction in external cause mortality [49, 50] —either directly (by reducing the number of young women with a critical shortage of capabilities to raise children) or indirectly (via the benefits of improved socioeconomic circumstance and capabilities that accrue over time). In addition, socioeconomic disadvantage is featured in the literature as being associated with accident, suicide and homicide mortality, and is more commonly experienced by Aboriginal populations [35].

In our data, the proportion of mothers less than 15 years was 60 times higher in Aboriginal women than non-Aboriginal women. Stress and socioeconomic disadvantage may actually prompt higher teenage pregnancy in Aboriginal women. In the *Western Australian Aboriginal Child Health Survey* [51], authors made the point:

*While most early teenage pregnancies are unplanned, there is a significant proportion of very disadvantaged young teenage girls who are motivated to proceed with the pregnancy to escape stressful family or school situations, to have a child to love, and to secure the financial means of living independently* (Page 181, [51]).

### Strengths and limitations

Our results were based on registry data with the advantages that they did not rely on recall and hence were more likely to be objective and uncensored. We used a recognised algorithm for the purposes of Aboriginal identification, and applied it to five variables of ethnicity obtained from four registries [15]. This meant that our accuracy of correctly identifying a woman’s Aboriginal status was optimised. We were able to access reliable demographic information for all women in our study population and reliable cause of death information from the Death Registry. The study data enabled us to estimate the number of surviving children of the women in our study population at either the end of the study period or the time of maternal death. This meant that we were able to accurately calculate and compare the number of children who experienced a maternal loss by the Aboriginal status of their mother. In a similar way, we were able to accurately calculate and compare the index parity of Aboriginal mothers who had died with those who had survived.

A limitation is that there are only area level measurements of SES and no individual measures which can be problematic if area level measures are interpreted at an individual level [52, 53]. Furthermore, in remote and other Aboriginal communities, status is measured by different things such as knowledge not acquired through our education system [54, 55]. Our calculation of the number of children in a mother’s care is only an estimate since we only knew the number of previous live births of each mother at the time of the birth of her last child during the study period. Women may have been caring for more or less than the number of their biological children depending on individual family arrangements. Moreover, the numbers for Aboriginal mothers are likely to be less reliable than the numbers for non-Aboriginal mothers since Aboriginal women more often care for the children of relatives [42].

There are data quality issues with Aboriginal mortality data since it may be difficult to determine the Aboriginal status of a deceased person since this may be done by a coroner and not a family member [1]. There is also recent evidence provided by the Registry of Births, Deaths and Marriages in Queensland that Aboriginal deaths are understated. Of 374 unregistered deaths, nearly 80 % were later found to be of Aboriginal people. This under-counting would reduce our HRs [56]. There are also concerns that death by external causes, and Aboriginal suicides in particular, are under-reported [57]. Other issues include distinguishing between intentional and accidental drug overdoses, falls and drownings [58]. Unfortunately, the ICD codes for homicide focus on the means of the homicide without mention of the relationship of the perpetrator to the victim. Details of the perpetrator, along with the location of the death (such as home, other residence, public place and so on) could further inform the development of preventive measures.

## Conclusions

This study found that Aboriginal mothers had a distinctly higher risk of death from external causes (accidents, suicides and homicides) than other Australian mothers. Our findings indicate that socio-demographic factors that are associated with premature mortality (such as lower SES [59, 60] and geographic remoteness) only partially account for the elevated risk of death from external causes among Aboriginal mothers. There are likely to be a range of other risk factors associated with the excess of potentially preventable accident, suicide and homicide deaths that also contribute to socio-demographic circumstance, but are not measured in the available data. Further investigation is required to understand the reasons for the disparity in external cause mortality between Aboriginal and other mothers and the possible causal mechanisms, including mental health issues, substance abuse, domestic violence, stress related to unemployment, loss of culture pervaded by social exclusion, racism and disrespect from mainstream society [60].

## Additional file

Additional file 1: Cause of death codes of diagnostic categories. (DOCX 16 kb)

## Abbreviations

Aboriginal: Aboriginal and Torres Strait Islander
ARIA: Accessibility/Remoteness Index of Australia
HMDS: hospital morbidity data system
HMDS: hospital morbidity data system
HRs: hazard ratios
MHIS: mental health information system
MNS: midwives notification system
SES: socioeconomic status
SES: socioeconomic status
WA: Western Australia
WADLS: WA data linkage system
