# Supplementary material for: Early mortality from external causes in Aboriginal mothers: a retrospective cohort study
Source: BMC Public Health. 2016 Jun 1;16:461. doi: 10.1186/s12889-016-3101-2 (PMC4888491; doi:10.1186/s12889-016-3101-2)
Supplement: Additional file 1: — Cause of death codes of diagnostic categories. (DOCX 16 kb) [file 12889_2016_3101_MOESM1_ESM.docx]

Supplementary Table 1: Cause of death codes of diagnostic categories

| Category | ICD-9 code | ICD-10 code |
| --- | --- | --- |
| *Any external cause* | **80-99, E870-E879, E930-E949, E980-E984, E985.5-E998** | **V01-Y98** |
| *Accidents* | **800-899, 90-94, 97-99** | **V01-X59, Y85-Y86** |
| *Transport* | 800-825 | V01-V99,Y85 |
| *Suicide* | **950-959** | **X60-X84,Y87.0** |
| *Hanging* | 953 | X70 |
| *Homicide* | **960-969** | **X85-Y09, Y87.1** |
| *Other external causes* | **E870-E879, E930-E949, E980-E984, E985.5-E998** | **Y10-Y84, Y87.2-Y98** |

Supplementary table 2: Number of children experiencing a maternal death by maternal Aboriginal status

| **Experiencing maternal death** | | | | | | **Not experiencing maternal death** | | | | | | | |
| --- | --- | --- | --- | --- | --- | --- | --- | --- | --- | --- | --- | --- | --- |
| **No children per mother** | **Non-Aboriginal**  **mothers** | **Total non-Aboriginal**  **children** | **Aboriginal**  **mothers** | **Total Aboriginal**  **children** | **Total mothers** | **Total**  **children** | **No children per mother** | **Non-Aboriginal**  **mothers** | **Total non-Aboriginal**  **children** | **Aboriginal**  **mothers** | **Total Aboriginal**  **children** | **Total mothers** | **Total**  **children** |
| 1 | 669 | 699 | 107 | 107 | 776 | 806 | 1 | 88,112 | 88,107 | 3,076 | 3,076 | 91,188 | 91,183 |
| 2 | 1,086 | 2172 | 115 | 230 | 1,201 | 2402 | 2 | 145,221 | 290,442 | 3,000 | 6,000 | 148,221 | 296,442 |
| 3 | 764 | 2292 | 144 | 432 | 908 | 2,724 | 3 | 79,481 | 238,443 | 2,808 | 8,424 | 82,289 | 246,867 |
| 4 | 322 | 1288 | 119 | 476 | 441 | 1764 | 4 | 28,588 | 114,352 | 2,385 | 9,540 | 30,973 | 123,892 |
| 5 | 129 | 645 | 95 | 475 | 224 | 1,120 | 5 | 8,796 | 43,980 | 1,535 | 7,675 | 10,331 | 51,655 |
| 6 | 49 | 294 | 62 | 372 | 111 | 666 | 6 | 3,007 | 18,042 | 1,004 | 6,024 | 4,011 | 24,066 |
| 7 | 27 | 189 | 37 | 259 | 64 | 448 | 7 | 1,177 | 8,239 | 554 | 3,878 | 1,731 | 12,117 |
| 8 | 10 | 80 | 17 | 136 | 27 | 216 | 8 | 415 | 3,320 | 281 | 2,248 | 696 | 5,568 |
| 9 | 5 | 45 | 6 | 54 | 11 | 99 | 9 | 201 | 1,809 | 136 | 1,224 | 337 | 3,033 |
| 10 | 2 | 20 | 6 | 60 | 8 | 80 | 10 | 93 | 930 | 67 | 670 | 160 | 1,600 |
| 11 | 1 | 11 | 5 | 55 | 6 | 66 | 11 | 48 | 528 | 30 | 330 | 78 | 858 |
| 12 | 0 | 0 | 0 | 0 | 1 | 0 | 12 | 24 | 288 | 7 | 84 | 31 | 372 |
| 13 | 0 | 0 | 1 | 13 | 1 | 13 | 13 | 8 | 104 | 3 | 39 | 11 | 143 |
| 14 | 0 | 0 | 0 | 0 | 0 | 0 | 14 | 3 | 42 | 4 | 56 | 7 | 98 |
| 15 | 0 | 0 | 0 | 0 | 0 | 0 | 15 | 1 | 15 | 1 | 15 | 2 | 30 |
| 18 | 0 | 0 | 0 | 0 | 0 | 0 | 18 | 0 | 0 | 1 | 18 | 1 | 18 |
| Total | 3,064 | 7,735 | 714 | 2,669 | 3,778 | 10,404 | Total | 355,170 | 808,641 | 14,892 | 49,301 | 370,067 | 857,942 |
